# Calcium-Activated Cl^−^ Channel: Insights on the Molecular Identity in Epithelial Tissues

**DOI:** 10.3390/ijms19051432

**Published:** 2018-05-10

**Authors:** Trey S. Rottgen, Andrew J. Nickerson, Vazhaikkurichi M. Rajendran

**Affiliations:** 1 Department of Physiology, Pharmacology, and Neuroscience, West Virginia University School of Medicine, Morgantown, WV 26506, USA; t.rottgen@gmail.com (T.S.R.); anicker2@mix.wvu.edu (A.J.N.); 2 Department of Biochemistry and Molecular Pharmacology, West Virginia University School of Medicine, Morgantown, WV 26506, USA

**Keywords:** TMEM16A, CLCA1, Cl^−^ channels, Ca^2+^, Ca^2+^-activated Cl^−^ channels, epithelium

## Abstract

Calcium-activated chloride secretion in epithelial tissues has been described for many years. However, the molecular identity of the channel responsible for the Ca^2+^-activated Cl^−^ secretion in epithelial tissues has remained a mystery. More recently, TMEM16A has been identified as a new putative Ca^2+^-activated Cl^−^ channel (CaCC). The primary goal of this article will be to review the characterization of TMEM16A, as it relates to the physical structure of the channel, as well as important residues that confer voltage and Ca^2+^-sensitivity of the channel. This review will also discuss the role of TMEM16A in epithelial physiology and potential associated-pathophysiology. This will include discussion of developed knockout models that have provided much needed insight on the functional localization of TMEM16A in several epithelial tissues. Finally, this review will examine the implications of the identification of TMEM16A as it pertains to potential novel therapies in several pathologies.

## 1. Introduction

Cellular Cl^−^ ion movement is involved in a vast array of physiological processes [1,2,3,4,5]. This includes the secretory function of practically any epithelial cell, transmission of sensory impulses, and smooth muscle contraction at various anatomical sites [1,2,3,4,5].

**Figure 1:**
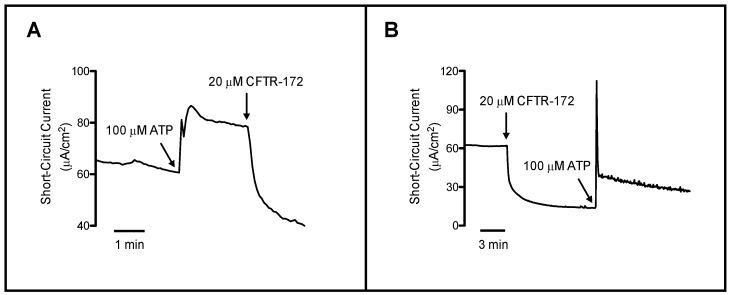
Presence of Cystic Fibrosis transmembrane conductance regulator (CFTR) and non-CFTR mediated Cl^−^ secretion in normal rat tracheal epithelium. Chloride secretion was measured as short-circuit current (I_SC_) in tracheal epithelia that were mounted under voltage clamp condition. (**A**) Effect of CFTR-172_inh_ (CFTR inhibitor; 20 μM) on ATP stimulated Cl^−^ secretion. Mucosal ATP (100 μM) addition stimulates Cl^−^ secretion that consists of two components—the sharp initial increase in I_SC_ immediately followed by a second, more rounded and prolonged current. Inhibition by mucosal CFTR-172_inh_ (CFTR inhibitor) indicates that the ATP-stimulated sustained Cl^−^ secretion is mediated by CFTR. The CFTR-172_inh_ inhibition of I_SC_ below baseline indicates that CFTR also accounts for a portion of basal Cl^−^ secretion in normal trachea; (**B**) Effect of ATP on Cl^−^ secretion in CFTR-172_inh_ pre-incubated trachea. In CFTR-172_inh_ pre-incubated trachea, mucosal ATP (which transiently increases intracellular Ca^2+^) transiently stimulates Cl^−^ secretion with a minimal plateau phase of Cl^−^ secretion. The transient Cl^−^ secretion represents non-CFTR mediated Cl^−^ secretion (i.e., through TMEM16A). The presence of a residual plateau phase of Cl^−^ secretion may likely be attributable to CFTR activation in response to ATP, which cannot be completely inhibited by 20 μM CFTR-172_inh_. (The unpublished data presented in this figure is in good agreement with the literature).

Previous work on different epithelial tissues has characterized Cl^−^ secretion to have primarily two different constituents—cyclic adenosine monophosphate (cAMP)-stimulated and Ca^2+^-stimulated Cl^−^ secretion [6,7] (Figure 1). Early research utilizing respiratory epithelium from Cystic Fibrosis (CF) patients was characterized by the absence of cAMP-stimulated Cl^−^ secretion [8]. However, those same tissues exhibited a significantly larger Ca^2+^-activated Cl^−^ conductance when administered Ca^2+^ ionophores, such as ionomycin or A23187 [7,9]. Also, cells isolated from a pancreatic tumor arising in a CF patient demonstrated a similar profile of Cl^−^ secretion to that of the respiratory epithelium (52). This then led to the understanding that the observed Ca^2+^-activated Cl^−^ secretion was serving a compensatory role in the absence of the cAMP-stimulated Cl^−^ secretion [8,10]. In 1991, Kartner et al. were able to demonstrate that expression of the CF gene in non-Cl^−^ secreting invertebrates (Sf9 cells) led to a cAMP-sensitive Cl^−^ conductance similar to that found in native healthy tissue [11]. However, the identity of the Cl^−^ channel mediating the Ca^2+^-activated Cl^−^ conductance observed in epithelial tissue from CF patients was still unknown. 

Following the major discovery of the Cystic Fibrosis transmembrane regulator (CFTR), many putative Ca^2+^-activated Cl^−^ channels (CaCC) have been examined [9,11]. In pursuing the molecular identity of the CaCC, a protein now referred to as CLCA1 (Cl^−^ channel accessory 1) received much of the early attention [12,13,14]. The initial cloning and isolation of this putative CaCC was from the bovine trachea (bCLCA1) [12]. This 125-kDa bCLCA1 protein, which was post-translationally modified to a 38-kDa protein, was able to yield macroscopic currents in *Xenopus* oocytes injected with the entire cRNA open-reading frame of the channel [12]. However, the suspected channel exhibited a sensitivity to 1 mM dithiothreitol (DTT; a reducing agent), which was not previously reported as an inhibitor of the native CaCC [12]. Also, when bCLCA1 was reconstituted into COS-7 cells, the observed current was insensitive to 100 µM niflumic acid (CaCC inhibitor), whereas the native CaCC was fully sensitive with an apparent half-maximal inhibitory concentration (K_i_) of 17 µM [12]. Ensuing discoveries of the murine (mCLCA1) and human homologs (hCLCA1) also described a post-translational processing that resulted in an approximate 39 and 90 kDa protein [15,16]. The two newly described homologs differed, however, from the bovine variant in that both putative channels exhibited sensitivity to niflumic acid [15,16]. However, similar to bCLCA1, the two variants demonstrated an inhibition when exposed to 2 mM DTT [15,16]. As previously mentioned, this was different from the originally described CaCC in *Xenopus* oocytes [17]. Also, the concentration of Ca^2+^ that was necessary to stimulate Cl^−^ channel opening was of supraphysiological concentrations (2 mM) [12,15,16]. The second human homolog [hCLCA2] to be described was similar in many attributes to the hCLCA1 counterpart [13]. Once again, very high concentrations of Ca^2+^ were required for activation, currents exhibited a sensitivity to DTT, and the lack of the previously observed time-dependence of activation seen in the native channel left concerns that the CLCA family was not the native CaCC [18]. Finally, several groups have published studies focusing on CLCA3 that demonstrated that the protein was secreted from cells and functioned as an extracellular protease [13,14]. This study, along with the observed functional differences from the native channel basically confirmed that the CLCA family was not the native CaCC, and the group of proteins was more likely extracellular proteases [12,13,14,15,16]. 

Recently, a new CaCC-designated TMEM16A has been characterized. TMEM16A was first described as a CaCC by three separate groups in 2008 [19,20,21]. As previously discussed, earlier candidate CaCCs did not demonstrate electrophysiological properties similar to the natively identified channel. However, TMEM16A was the first CaCC that demonstrated a Ca^2+^-activation that matched that of the native channel found in many tissues [20]. Yang et al. demonstrated a slight-voltage dependence of TMEM16A at submicromolar and low micromolar concentrations of Ca^2+^, which was similar to the native protein [21]. This effect was illustrated by greater channel activation at more depolarized potentials (+60 mV vs. −60 mV) in transfected human embryonic kidney 293 (HEK 293) cells with varying Ca^2+^ concentrations [21]. Also, the group utilized small-interfering RNA (siRNA) injected intravenously in mice targeted to TMEM16A transcript to elucidate the role the channel may play in secretion of saliva [21]. Pilocarpine (muscarinic agonist) stimulated saliva secretion was significantly inhibited with a corresponding decrease in TMEM16A immunostaining in submandibular glands [21]. Caputo et al., also utilizing siRNA targeted to TMEM16A mRNA, transfected confluent monolayers of primary human bronchial epithelial cells [19]. Treated monolayers demonstrated significant decreases in UTP-stimulated (Ca^2+^-activated, via membrane G_αq_-coupled purinergic receptors) short-circuit current [I_SC_] [19]. This study further established the possibility of this specific CaCC being not only ubiquitous, but also the long sought-after native CaCC [19].

## 2. TMEM16A Characterization

Following these initial discoveries outlined above, considerable research has focused on the physical characterization of TMEM16A [22,23,24]. One of the initial findings suggested that the final quaternary structure of TMEM16A existed as a dimer in the plasma membrane [25]. This was demonstrated with TMEM16A proteins that were fused to either GFP or mCherry [25]. The different TMEM16A conjugates were able to undergo fluorescence resonance energy transfer (FRET), which indicated a close proximity of the two proteins [25]. However, it was not until a couple of years later that the actual sequence of amino acids important for this interaction was identified [26]. Mutants of TMEM16A lacking an α-helix that corresponded to residues 161–179 were not able to form functional channels, hence the lack of observed Cl^−^ currents in transfected HEK 293 cells [26]. 

**Figure 2:**
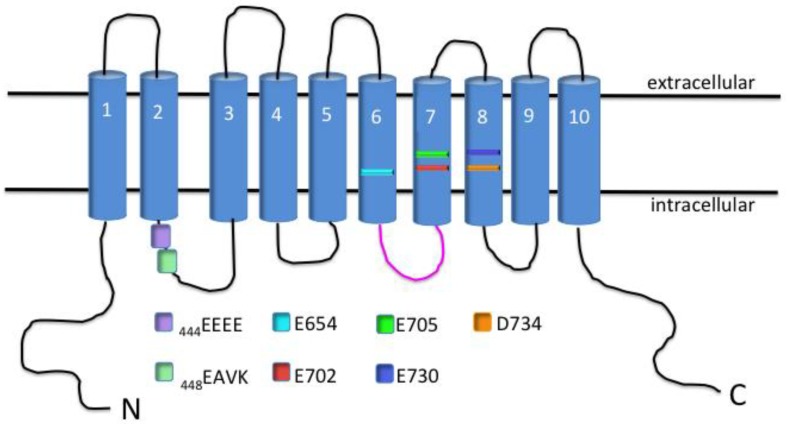
Schematic of TMEM16A channel protein with identified residues necessary for voltage and Ca^2+^-sensitivity. TMEM16A channel protein consists of 10 transmembrane domains (TMD). The intracellular loop between TMD-2 and TMD-3 contains the voltage-sensitive _444_EEEE residues, as well as the _448_EAVK residues that participate in Ca^2+^-sensitivity of the channel. The E702 and E705 residues located within the seventh TMD also participate in Ca^2+^-sensitivity of the channel. The third intracellular loop (shown in pink), and the residues E654, E730 and D734 have also been implicated to affect Ca^2+^-sensitivity of the channel.

Shortly after this discovery, a group was able to identify several residues that were important for the voltage-dependence of the channel, as well as amino acids that participate in the Ca^2+^-sensitivity of TMEM16A [27]. The residues that confer a voltage-dependence of the channel were found to be located within the first intracellular loop and consisted of four repeating glutamic acid residues [_444_EEEE] (Figure 2) [27]. Alanine substitution at these residues shifted the half-maximal activation of the channel from 64 ± 0.9 mV to a more depolarized potential of ≈160 mV at 1 μM Ca^2+^ [27]. However, the residues important for Ca^2+^-sensitivity were found to be located directly adjacent to the glutamic acid residues and consisted of a glutamic acid, alanine, valine and lysine [_448_EAVK] (Figure 2) [27]. Deletion of these residues was able to shift the Ca^2+^-sensitivity drastically from 1 μM (which was able to increase open-probability at very hyperpolarized potentials) to 25 μM Ca^2+^ (which could increase open-probability only marginally) [27]. While EAVK residues are undoubtedly important for channel gating, it has also been shown that glutamic acid residues E702/705 are also essential for Ca^2+^-sensitivity of TMEM16A (Figure 2) [28]. This was demonstrated by mutants of these two residues having a Ca^2+^-sensitivity several orders of magnitude less than their respective wild type (WT) channel [Ca^2+^], 20 μM WT vs. 2 mM E702/705 mutant [28]. The importance of these residues was confirmed by another group that mutated these same amino acids and obtained a channel with significantly less Ca^2+^-sensitivity [29]. This group was able to demonstrate cooperativity between these residues and the amino acids initially found to confer sensitivity [_448_EAVK] [29]. Three other acidic moieties [E650, E730, D734] have also been shown to contribute to the Ca^2+^-sensitivity of TMEM16A [28]. Also, Scudieri et al. developed TMEM16A chimera proteins by substituting residues from TMEM16B [another member of the protein family, also exhibiting Ca^2+^-stimulated Cl^−^ secretion] into the sequence of TMEM16A to determine potential domains necessary for Ca^2+^-binding [30]. The group was able to determine from their results that the third intracellular loop of TMEM16A participates in conferring Ca^2+^-sensitivity to the channel [30]. This was made obvious by the deletion of these residues resulting in a shift of the EC_50_ of Ca^2+^ from 0.25 μM to 2 μM at a holding potential of +100 mV [30]. 

Besides activation of the channel, Ca^2+^-binding has also previously been shown to affect anion permeability ratios. Peters et al. was able to show that lower concentrations of intracellular Ca^2+^ (400 nM) was able to elicit an approximate 5:1 Iodide (I^−^):Cl^−^ permeability ratio, while higher concentrations of Ca^2+^ were smaller in magnitude (≈3:1, I^−^:Cl^−^). Thiocyanate (SCN^−^) substitution demonstrated a similar qualitative profile to that of I^−^:Cl^−^ permeability; however, SCN^−^ was larger in magnitude (≈12:1, SCN^−^:Cl^−^) [31].

While the previously mentioned work was essential for identifying residues necessary for Ca^2+^-binding and voltage-sensitivity [27,28,30], it was not until the crystal structure was elucidated that researchers could more clearly visualize the interaction of the previously mentioned residues [and several others] in Ca^2+^-binding and channel gating of TMEM16A [32]. In 2014, Brunner et al. were the first group to generate a crystal structure of TMEM16 from *Nectria haematococca* [nhTMEM16] [32]. The conserved protein from *Nectria haematococca* only functions as a lipid scramblase; however, the protein is still sensitive to Ca^2+^ and demonstrates increased scramblase activity with increasing concentrations of Ca^2+^ [32]. The results of the study were able to demonstrate that nhTMEM16 does in fact associate as a dimer in the plasma membrane [32]. This study also described a Ca^2+^-binding segment that was embedded within the hydrophobic membrane [32]. This research group was also the first one to postulate the possible mechanisms of ion conductance by either a single-pore or double-barreled architecture [23,32]. Following the initial observations obtained from the crystal structure of nhTMEM16, two different groups were able to resolve the crystal structure of TMEM16A from murine origin [24,33,34]. This new information about the channel was able to illuminate important residues for interaction with conducting anions, as well as to illustrate that each monomer of TMEM16A was able to bind two individual Ca^2+^ ions [33]. Also, the greater resolution with the murine TMEM16A allowed for an accurate description of how Ca^2+^-binding mediates Cl^−^ conductance [34]. This is accomplished via a hinge mechanism that is dependent on a glutamic acid residue [E654] interacting with two Ca^2+^ ions that allow for opening of a single pore within the channel [34]. 

Associated proteins and β-subunits have been shown time and time again to be critical in the functioning of a plethora of ion channels [35,36,37]. TMEM16A is no exception, with several reports claiming the protein calmodulin [CaM] to associate with the channel and be “indispensable” to the channel’s function [38,39]. The initial report utilized murine TMEM16A transfected into HEK 293 cells for whole-cell patch clamp experiments [38]. The group was able to show that whole-cell conductance stimulated by ionomycin was significantly attenuated [Δ*G*_iono_, 45 ± 5.2 nS vs. 17 ± 2.1 nS] when a specific inhibitor of CaM [trifluoperazine, 10 μm] was present in the pipette solution [38]. Another group soon followed with a report of CaM affecting the relative permeability of different anions [Cl^−^, HCO_3_^−^] [39]. When CaM was exogenously added to cytosolic portion of the membrane, HCO_3_^−^ permeability markedly increased [0.39 ± 0.09 to 0.97 ± 0.06 AU] [39]. However, several recent studies dispute the importance of CaM’s interaction with TMEM16A [40,41]. Terashima et al. was able to purify human TMEM16A and reconstitute the protein in liposomal membranes [42]. The channel was directly activated by Ca^2+^ with an approximate EC_50_ for Ca^2+^ of 210 nM [42]. And when the group reconstituted the protein with CaM, they were not able to observe an association of the two proteins or any shifts in Ca^2+^-sensitivity [42]. Another group overexpressed a dominant-negative form of CaM with TMEM16A [40]. Whole-cell patch clamp experiments from the study did not demonstrate a difference in total current measured or changes in half-maximal activation of the channel when performed under conditions with the dominant-negative CaM [40]. Finally, a group from the University of California-Davis directly disputed the previously published results of the effects of CaM on TMEM16A permeability to different anions [41]. In all, the importance of CaM on TMEM16A function is most certainly divided within the field [38,40]. However, it is known that TMEM16A does have putative binding sites for CaM, but the importance of that interaction is still under intense discussion and research.

Another protein that has been implicated to interact with TMEM16A is the extracellular protease CLCA1 [43,44,45]. CLCA1 originally was thought to be a CaCC itself, as discussed above. However, it is now accepted that the protein partially functions to modulate TMEM16A membrane expression and function [44]. Co-culture of HEK 293 cells expressing TMEM16A with cells that actively secrete CLCA1 was able to elicit large, outwardly rectifying, Ca^2+^-sensitive currents, while TMEM16A cells co-cultured with empty vector only exhibited modest increases in current [45]. Also, a group has shown that the von Willebrand factor domain of CLCA1 is responsible for the increase in observed currents during patch clamp electrophysiology [44]. Published research on CLCA1 and its interaction with TMEM16A is much more limited. However, all seem to point to an increase in overall membrane expression with a potential to increase the actual conductance of TMEM16A [43,44,45]. 

## 3. TMEM16A in Epithelial Tissues

While research continues to characterize the channel and its potential interactions, one of the initial observations describing TMEM16A as a CaCC demonstrated its potential physiological importance [19,21]. Yang et al. was able to show a lack of pilocarpine-stimulated Ca^2+^-activated Cl^−^ secretion in salivary secretory epithelium in the mouse following siRNA-mediated TMEM16A knockdown [21]. The importance of this discovery was not simply related to the identification of the native CaCC protein in salivary epithelium, but also demonstrated the importance of Ca^+^-activated Cl^−^ secretion in the process of saliva production [21]. Following this preeminent discovery, several groups followed with studies focusing on different epithelial tissues [46,47]. 

### 3.1. Respiratory Epithelium

**Figure 3:**
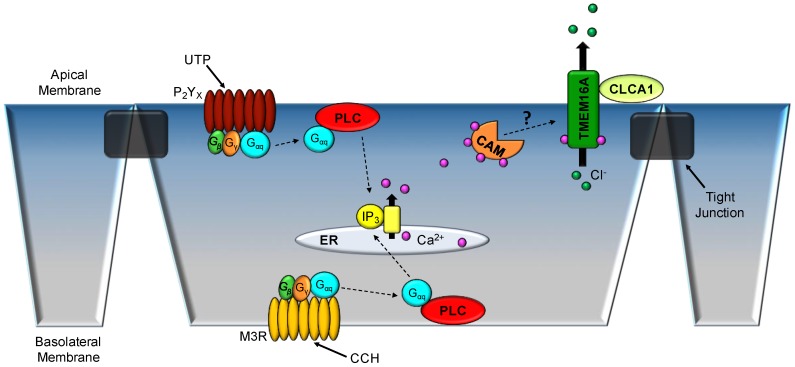
Cellular model of Ca^2+^-activated Cl^−^ secretion in tracheal epithelium. Either apical ATP or basolateral carbachol (CCH; a muscarinic receptor (M3R) agonist) administration transiently increases intracellular Ca^2+^ stores from endoplasmic reticulum (ER), leading to increased Ca^2+^-activated Cl^−^ secretion through TMEM16A. G-protein (Gαq)-coupled receptors, including the apical P_2_Y_X_ purinergic receptor (UTP) and basolateral M3R mediate the release of internal Ca^2+^ stores. Either Ca^2+^-calmodulin (CAM) or the extracellular protease CLCA1 may modulate TMEM16A mediated Cl^−^ secretion by activating the channel and/or increasing the plasma membrane expression. CLCA1—Cl^−^ channel accessary-1; PLC—phospholipase C.

Prior to the actual identification of TMEM16A as a CaCC, it was shown that disruption in the gene resulted in tracheomalacia, which was lethal within the first few days of life of neonatal mice [48]. Following the discovery of TMEM16A as a CaCC, Ousingsawat et al. isolated tracheas from the global TMEM16A knockout mice [neonates] for Ussing chamber studies of Ca^2+^-activated Cl^−^ currents [49]. Response to pharmacological agonists, such as ATP and CCH, which elicit release of intracellular Ca^2+^ (and normally increase apical Cl^−^ conductance) was significantly attenuated in the global knockout mice [49], thus indicating that TMEM16A plays a major role in the Ca^2+^-activated Cl^−^ secretion observed in WT mouse trachea. Residual stimulated-I_SC_ was shown through the use of pharmacological inhibitors to be mediated by the CFTR Cl^−^ channel [49]. However, the presented results of the publication were from neonatal mice suffering from multiple organ failures, which could be a confounding error of that study [49]. Several years after the original study, the group managed to generate animals that were lacking TMEM16A specifically in ciliated respiratory epithelium using the *FOXJ1* promoter [50]. Once again, murine tracheas were mounted in Ussing chambers and administered 100 μM ATP to the apical membrane (Figure 3) [50]. The elicited Ca^2+^-activated Cl^−^ currents were significantly attenuated in the transgenic animals [50]. Conventional whole-cell patch clamp electrophysiology of respiratory epithelium demonstrated a similar response when exposed to extracellular ATP [50]. 

Even the initial publications on TMEM16A demonstrated the channel to be present in polarized human bronchial epithelial cells [19]. Caputo et al. transfected cells with siRNA directed against the mRNA for TMEM16A, which resulted in significantly decreased I_SC_ in human bronchial epithelial cells [19]. Many studies have followed utilizing different respiratory cell lines that have also characterized the presence of TMEM16A [51,52]. 

More importantly, cell culture of primary respiratory epithelium, as well as immortalized cell lines, has demonstrated the significance of TMEM16A in respiratory pathology. The cytokine interleukin-4 (IL-4) has been well characterized to up-regulate protein expression of TMEM16A in respiratory epithelium [19,53]. IL-4 is also a major player in respiratory pathologies such as chronic obstructive pulmonary disease (COPD) and asthma [54,55]. Immunofluorescence of respiratory epithelium isolated from asthma patients clearly demonstrates an increase in TMEM16A expression [56,57]. Also, activation of TMEM16A with apical ATP in primary cultures of human bronchial epithelium seems to regulate secretion of mucin, one of the major hallmarks of inflammatory airway disease [56]. Taken together, targeted therapies against TMEM16A in diseases of airway inflammation could one day be a cornerstone of the treatment regimen.

On the other side, TMEM16A has potentially provided a new therapeutic target for the treatment of CF [10]. Previously published work using the *cftr*^−/−^ mice demonstrated only a mild pathology, which was in opposition to the observed disease in humans [10]. Organs that had minimal pathology compared to the human counterpart were observed to have substantial amounts of Ca^2+^-activated Cl^−^ secretion [10]. The preserved Cl^−^ secretion prevented severe pathology from developing within the lung [10]. With that in mind, much research has focused on increasing function or expression of TMEM16A in human tissues to help mitigate symptoms of CF or potentially reverse some of the associated pathology of CF [19,58]. 

### 3.2. Colonic Epithelium

Similar to the initial reports of TMEM16A-mediating the Ca^2+^-activated Cl^−^ current in respiratory epithelium, the neonatal mice were also used to study colonic epithelium [49,59]. Transepithelial potential [V_TE_] of distal colon administered basolateral carbachol (CCH; muscarinic agonist that increases intracellular Ca^2+^ concentration) was able to significantly hyperpolarize in *Tmem16a*^+/+^ mice [49]. However, *Tmem16a*^−/−^ littermates were not able to respond to basolateral CCH administration [49]. Calculated I_SC_ from control animals was approximately 60 μA/cm^2^, while *Tmem16a*^−/−^ animals had a calculated I_SC_ of about 10 μA/cm^2^ [49]. It was not until recently that the tissue-specific [*Vil1*] knockout mice of TMEM16A confirmed the previously observed results [50]. Similar to the global knockout mice, CCH-stimulated I_SC_ was significantly less in the *Tmem16a*^−/−^ animals [50]. 

TMEM16A has been observed in several human colonic epithelial cell lines. The HT-29 and T84 cell lines have both demonstrated expression of TMEM16A, characterized by immunoblot [60,61]. However, a group that employed the use of siRNA targeted against TMEM16A in the T84 cell line did not demonstrate a large decrease in ATP-stimulated I_SC_ [62]. The only change in the measured I_SC_ was the initial peak prior to the plateau phase of the trace, which would potentially indicate a minor role for TMEM16A in human colonic epithelium [62]. However, ATP-stimulated I_SC_ in T84 has previously been characterized to be mediated more through adenosine receptors, instead of Ca^2+^-increasing purinergic receptors [63].

As far as pathology related to TMEM16A in colonic epithelium, less is known partially due to an incomplete knowledge as to the expression of the channel in human colon. However, previous studies have shown that rotaviral infection in children causes diarrhea by increasing Ca^2+^-activated Cl^−^ secretion [64,65,66]. Ousingsawat et al. was able to show that NSP_4_, a synthetic peptide similar to a transcribed rotaviral peptide, worked through the activation of TMEM16A in the murine distal colon to increase Cl^−^ secretion [66]. Several studies have also indicated a potential role that the channel may play in the evolution of colon cancer [67,68]. TMEM16A has been observed to participate in apoptosis and up-regulation of the channel can increase growth and invasion of tumors [69,70,71]. There has also been some research that TMEM16A may participate in inflammatory bowel disease; however, even less is known about that, especially in regard to the mechanism by which TMEM16A may participate in the development of disease [72]. 

Potential therapies related to TMEM16A in colonic epithelium are far from clinical utility, especially with many questions still unresolved as to how the CaCC participates in these very complex diseases. With that said, small molecule inhibitors could potentially one day have utility in colon cancer treatment as adjunct therapy to the main course of action. As far as inflammatory bowel disease, so little is known about TMEM16A and its potential interaction that it would be pure speculation at this point. 

While the majority of this section has been focused on respiratory and colonic epithelium, it is worth noting that TMEM16A has been characterized as the CaCC in several other epithelial tissues [73,74,75]. Cell lines of pancreatic ductal cells have been observed to express TMEM16A [73,75]. Also, biliary epithelium from murine, rat and human origin has functionally been shown to express TMEM16A [46]. Finally, the presented collective knowledge here is not an all-inclusive list as many other epithelial tissues are likely to express TMEM16A and may be of future research endeavors. 

## 4. Conclusions

The CaCC, TMEM16A, is the native protein that is ubiquitously expressed across a wide variety of epithelial tissues. While the structure and function of the channel have been for the most part elucidated, much is still unknown about potential protein–protein interactions. These interactions could shed light on the possible cellular function that the channel may play in complex diseases such as cancer and inflammatory airway diseases. Continued research on therapies to increase protein expression of TMEM16A in the plasma membrane of CF patients is still under intense investigation, and hopefully one day will be an important part of ameliorating CF symptoms and associated-pathology. Also, as previously mentioned, TMEM16A may play a role in the development of inflammatory bowel disease. Further understanding of this potential role could provide insight into the overall development of the pathology, as well as provide novel therapies for treatment of the disease. Unfortunately, at this time there are not currently any treatments ready for clinical utility, but this highlights the need for continued discovery of the role and function of TMEM16A in a myriad of different pathologies. Hopefully with continued research, targeted therapies can be translated to clinical use in a timely manner for the benefit of a vast patient population.

## Abbreviations

ATP	adenosine triphosphate
CaCC	calcium-activated chloride channel
CaM	calmodulin
cAMP	cyclic adenosine monophosphate
CCH	carbachol
CF	Cystic Fibrosis
CFTR	Cystic Fibrosis Transmembrane Conductance Regulator
CLCA	Cl^−^ channel accessory
cRNA	complementary ribonucleic acid
DTT	dithiothreitol
EC_50_	half-maximal effective concentration
FRET	fluorescence resonance energy transfer
GFP	green fluorescent protein
HEK 293	human embryonic kidney 293 cells
IL-4	interleukin 4
I_SC_	short-circuit current
kDa	kilodalton
mM	millimolar
mRNA	messenger ribonucleic acid
mV	millivolts
µM	micromolar
siRNA	small interfering ribonucleic acid
TMEM16A	transmembrane member 16A
UTP	uridine triphosphate
V_TE_	trans-epithelial voltage
WT	wild type
